# Salicylic Acid in Pathologic Conditions of the Liver

**Published:** 1907-09

**Authors:** 


### Salicylic Acid in Pathologic Conditions of the Liver.

Charles Gilbert Davis, M.D., Chicago, Professor of Surgery and Gynecology, Lakeside Post-graduate School, reports (*Thera-*

*peutic Gazette,* July 15, 1907), on the treatment of catarrhal cholangitis and cholelithiasis with pills similar to Bauermeister's probilin, that is, consisting of acid sodium oleate, salicylic acid, phenolpthalein and menthol.

The use of salicylic acid in pathologic conditions of the liver he began twenty-five years ago, and he found it particularly satisfactory in combination with the other drug, as a cholagogue and an antiseptic whose effect is prolonged throughout the alimentary tract. Many cases which are ordinarily considered amenable only to surgical interference can be satisfactorily treated with the pills, and he believes that under their use cholangitis, with and without stones, will ultimately cease to be a surgical condition.

He relates six typical cases in which he found the combination effective, not as a purgative pill, but more especially as a cholagogue, a concretion solvent and a biliary disinfectant. The menthol and the phenolphthalein produce and regulate intestinal activity, and the salicylic acid and the oleate have a decided antiseptic and powerful cholagogue action.

As to diet, he does not restrict the same too closely, but interdicts all foods known to be difficult of digestion, and all alcoholic beverages. The principal point to be observed is to insist that large draughts of hot water be taken with the pills, for the purpose of diluting the excretions and assisting in breaking up any concretions present.

